# Supplementary material for: End-of-life experiences in individuals with dementia with Lewy bodies and their caregivers: A mixed-methods analysis
Source: PLoS One. 2024 Aug 29;19(8):e0309530. doi: 10.1371/journal.pone.0309530 (PMC11361593; doi:10.1371/journal.pone.0309530)
Supplement: S1 File — This file provides the semi-structured interview used in the study. The current analysis presents mixed-methods data pertaining to interview question #1. (PDF) [file pone.0309530.s002.pdf]

End of Life Semi-Structured Interview Questions (version 9.28.2021)

Semi-Structured Interview:

1. "Tell me about you and [name's/your loved one's] experience ***when he/she was near the end of life***." [Question about pre-death experiences.]

(Possible prompts – *you do not need to ask all these questions*:

For example, what symptoms seemed worse over that time?

What went well during the last few weeks or months of [name's] life?

What could have made that time of life easier or better?

What decisions had to be made before your loved one died? For example, what decisions needed to be made about whether or you or your loved one could remain at home, or whether you did or did not want to work with hospice?

What were your experiences with hospice?

Looking back, are there any things you would do differently about how you approached this time in your and [name's/your loved one's] life?)

2. "Can you tell me about your experiences and emotions in the last few months ***since [name] died***?" [Question about post-death experiences]

(Possible prompts: For some caregivers of people with dementia, the death of their loved one results in a mix of both grief and relief, or other mixed emotions. What has been your experience?)

Ending Question:

"We are coming to the end of the interview. What are the most important things that you would like others to know in order to help improve the end-of-life experience for other people with dementia with Lewy bodies and their families?"
