# Supplementary material for: End-of-life experiences in individuals with dementia with Lewy bodies and their caregivers: A mixed-methods analysis
Source: PLoS One. 2024 Aug 29;19(8):e0309530. doi: 10.1371/journal.pone.0309530 (PMC11361593; doi:10.1371/journal.pone.0309530)
Supplement: S2 File — This file provides the COREQ checklist for reporting qualitative data. (DOCX) [file pone.0309530.s003.docx]

S2 File. COREQ 32-item checklist for **“**End-of-Life Experiences in Individuals with Dementia with Lewy Bodies and their Caregivers: a Mixed-Methods Analysis”

| **No. Item** | **Guide questions/description** | **Reported on Page #** |
| --- | --- | --- |
| **Domain 1: Research team and reﬂexivity** |  |  |
| *Personal Characteristics* |  |  |
| 1. Interviewer/facilitator | Which author/s conducted the interview or focus group? | 7-8 (coordinators; only KS and HF are co-authors) |
| 2. Credentials | What were the researcher’s credentials? E.g. PhD, MD | 7-8 (coordinators) |
| 3. Occupation | What was their occupation at the time of the study? | 7-8 (coordinators) |
| 4. Gender | Was the researcher male or female? | N/A (various coordinators and gender not requested/assessed) |
| 5. Experience and training | What experience or training did the researcher have? | 8 (trained as part of study) |
| *Relationship with participants* |  |  |
| 6. Relationship established | Was a relationship established prior to study commencement? | 7-8 (coordinators) |
| 7. Participant knowledge of the interviewer | What did the participants know about the researcher? e.g. personal goals, reasons for doing the research | N/A (interviews done at final visit of ongoing study; study goals discussed long before interview portion) |
| 8. Interviewer characteristics | What characteristics were reported about the interviewer/facilitator? e.g. Bias, assumptions, reasons and interests in the research topic | N/A (interviews done by coordinators at final visit of ongoing study; research topic discussed at initial approach & screening visits) |
| **Domain 2: study design** |  |  |
| *Theoretical framework* |  |  |
| 9. Methodological orientation and Theory | What methodological orientation was stated to underpin the study? e.g. grounded theory, discourse analysis, ethnography, phenomenology, content analysis | 8-9 (thematic analysis) |
| *Participant selection* |  |  |
| 10. Sampling | How were participants selected? e.g. purposive, convenience, consecutive, snowball | 5-6 |
| 11. Method of approach | How were participants approached? e.g. face-to-face, telephone, mail, email | 5-6 |
| 12. Sample size | How many participants were in the study? | 6-7, 9 |
| 13. Non-participation | How many people refused to participate or dropped out? Reasons? | N/A (first 50 post-death interviews) |
| *Setting* |  |  |
| 14. Setting of data collection | Where was the data collected? e.g. home, clinic, workplace | 7 (study partner at home, via phone or Zoom) |
| 15. Presence of non-participants | Was anyone else present besides the participants and researchers? | N/A (caregivers were at home; they were not asked if other people were in house at time of interview) |
| 16. Description of sample | What are the important characteristics of the sample? e.g. demographic data, date | 9-12 |
| *Data collection* |  |  |
| 17. Interview guide | Were questions, prompts, guides provided by the authors? Was it pilot tested? | 7, S1 File |
| 18. Repeat interviews | Were repeat interviews carried out? If yes, how many? | N/A (no) |
| 19. Audio/visual recording | Did the research use audio or visual recording to collect the data? | 8 |
| 20. Field notes | Were ﬁeld notes made during and/or after the interview or focus group? | N/A (no) |
| 21. Duration | What was the duration of the interviews or focus group? | 7 |
| 22. Data saturation | Was data saturation discussed? | 7, 8 |
| 23. Transcripts returned | Were transcripts returned to participants for comment and/or correction? | No |
| **Domain 3: analysis and ﬁndings** |  |  |
| *Data analysis* |  |  |
| 24. Number of data coders | How many data coders coded the data? | 8-9 |
| 25. Description of the coding tree | Did authors provide a description of the coding tree? | Results, S4 File |
| 26. Derivation of themes | Were themes identiﬁed in advance or derived from the data? | 8-9 |
| 27. Software | What software, if applicable, was used to manage the data? | 9 (ATLAS.ti) |
| 28. Participant checking | Did participants provide feedback on the ﬁndings? | 9 (no) |
| *Reporting* |  |  |
| 29. Quotations presented | Were participant quotations presented to illustrate the themes/ﬁndings? Was each quotation identiﬁed? e.g. participant number | Results, S4 File |
| 30. Data and ﬁndings consistent | Was there consistency between the data presented and the ﬁndings? | Results. S4 File |
| 31. Clarity of major themes | Were major themes clearly presented in the ﬁndings? | Results, S4 File |
| 32. Clarity of minor themes | Is there a description of diverse cases or discussion of minor themes? | Results, S4 File |
