# Supplementary material for: End-of-life experiences in individuals with dementia with Lewy bodies and their caregivers: A mixed-methods analysis
Source: PLoS One. 2024 Aug 29;19(8):e0309530. doi: 10.1371/journal.pone.0309530 (PMC11361593; doi:10.1371/journal.pone.0309530)
Supplement: S3 File — This file provides the qualitative analysis codebook for the current analysis including the primary contributing transcript excerpts to supplement the quotes provided in the manuscript. (DOCX) [file pone.0309530.s004.docx]

**S4 File. Codebook for “End-of-Life Experiences in Individuals with Dementia with Lewy Bodies and their Caregivers: a Mixed-Methods Analysis”**

**RESEARCH QUESTION 1 (RQ1):** What are the experiences of individuals with DLB and their caregivers near the end of life of the person with DLB?

1. **THEME LABEL: MOTOR (N = 33)**

**Thematic properties:**

- - **Eating/feeding**: Assistance with eating activities (e.g., cutting up food, being served soft/easy to swallow foods, putting silverware in their hands or help using silverware) but still independently feeding themselves with assistance, still has appetite.
  - **Toileting/bathing:** Incontinence, trouble bathing independently, getting assistance with grooming (e.g., brushing hair, teeth, getting dressed).
  - **Mobility:** trouble with balance, walking, coordination, getting to and from places themselves physically, etc. May use a wheelchair, walker, cane, etc. or have caregivers pick them up and place them.
  - **Speech:** Trouble speaking or choosing not to speak.

| **Properties** | **Representative/Exemplar Illustrations - Data Excerpts** | **N** |
| --- | --- | --- |
| Eating/feeding | I said, “I'll feed you.” He said, "I can." He reached for his fork, and I said, “why don't I feed you because you're not going to enjoy sleeping with green beans on the bed.” We used to laugh about ridiculous things, and so he let me feed him, and he ate. [1927-35]  “She always had a very healthy appetite, and she still did, but she was having problems getting food to her mouth. I ended up, probably the month before I would have to help her eat… she shook a lot. She didn't shake up until the month before she passed. She shook a lot. I mean, the food would fall off her pork or spoon before she puts it in her mouth.” [1927-17]  “I think in the last three months, definitely he was in a steady decline … it was harder for him to eat on his own. I had to start feeding him, more and more consistently as well. He might have a day where he could actually pick up the utensils and feed himself. And then some days he just wouldn't. It got to the point in the last couple of months where he had so little energy and he wouldn't have his eyes open, but he would eat if I fed him. Like, he would respond, and he would chew and he would swallow and stuff, but he just had no energy.” [1927-100]  “I ended up hav[ing] to feed him. At first, it was one of those, well, I'll just cut your meat for you, kind of a thing. But you have to feed the person that you love most in the whole world, which is not that you mind doing it, but he hated it. He hated that I had to feed him.” [1923-06] | 12 |
| Toileting/bathing | The incontinence was a lot to deal with, but the dementia wasn't so bad that he couldn't keep his depends onhe knew the number one rule in this house was to wear depend. [1927-44]  I had to wash his hair and dry it, and I would dry him off help him get dressed. [1926-01]  The week before…we backed off trying to feed him. He clearly had lost his appetite. [We] backed off trying to get him to the bathroom did sponge baths as opposed to taking him into the bathroom. [1927-23]  “I dressed him every day and I showered him twice a week. I shaved him every other day.” [1926-09] | 11 |
| Mobility | When he started losing his ability to, well not being able to walk was a big deal. And then he started having balance problems even sitting in a chair, he would tip to one side or the other and of course the lack of his fine motor movement, that was a problem too. Those symptoms were -- it just made it very difficult because he couldn't do all the things that he always did. [1927-10]  She wouldn't get up because any time she did, she could only walk about two feet. [1927-14]  The worst thing was that he no longer could get up. He fell and then he just couldn't hold himself up anymore. [1927-21]  “He went downhill that [last] month and just everything got harder. Walking got harder, sitting down got harder, getting up got harder.” [1927-75]  “In high sight, being what it was, I would have tried to involve the doctor sooner so that I could have had the care she needed that last month. I mean, and knowing now what I know, I would have contacted the doctor by the day she quit walking. I didn't contact her physician because I thought maybe she was sick [and that’s why she] had stayed in bed... So I guess I was expecting her to wake up and walk again and that did not happen.” [1927-17]  “The first week that she was there [at nursing home], she fell out of bed and went to the emergency room… then the same thing happened the next Friday. She fell out of bed in the evening, and all [they] sent her to the emergency room.” [1927-22]  “He fell that morning. He got up when I had dozed off and he fell face down, gashed, broke the nose, eyebrows. That was a Sunday morning. And so, I called hospice, and they came… he wasn't steady and he couldn't walk by himself, but he was trying, and I knew I couldn't do it by myself. So I said, "Let's take him there [to the hospice center].” [1927-77]  “She was getting to the point where she was going to need a two person assist. The last time I -- one of the last times I saw her, she needed two people to get her from the wheelchair that she was sitting in to a chair, an easy chair, which was like a foot away, not even. So, I was pretty worried. I was like, ‘oh my God, what's going to happen now? Is she going to fall some more? Is she going -- is she going to be bedridden?’ So I was really relieved to tell you the truth that it didn't -- there wasn't a horrible incident.” [1927-68] | 21 |
| Speech | The Parkinson's started taking away her voice. It was hard to get her to say anything and when she said anything, it was hard to hear her because her voice had become so soft. [1927-83]  [She] lost the ability to smile. And at that point she couldn't carry on a conversation. When she did try to it was just gibberish you what they call word saladnothing made sense. [1927-54]  I don't know if he was aware of how close he was to death, but he was aware that he was dying and he hated that [laughs]. He hated not being able to talk about it because his word searching was terrible. [1924-10]  “He couldn't really talk and make sense when he talked. I know he still understood what I was saying.” [1923-01]  “He wasn't really conversational probably for the past year. I mean, anything that was, you know, that he really thought, could think about, or ask questions, he just didn't.” [1927-59]  “She just had lost a good bit in the end. And, uh, so she wasn't very communicative, And, um, but we still, uh, I would take her out in the wheelchair, we'd wheel outside and go around the park… I can't pinpoint a [exact] time, I can't say it was [within the last] three months or that, but she did become less sort of aware. You know, wasn't talkative, and she wasn't facially communicating as much.” [1927-22] | 8 |

1. **THEME LABEL: COGNITIVE/BEHAVIORAL/PSYCHIATRIC SYMPTOMS (N = 29)**

**Thematic properties:**

- **Hallucinations/delirium:** Seeing things that aren’t there, mistaking objects for something else (e.g., thinking their cat is a child), being temporarily ‘out of it’ (e.g., “zombie mode”).
- **Memory decline / memory loss:** including trouble with cognition, language processing, general awareness of surroundings, confusion, etc.; losing ability to process information (which may relate to ‘speech’ under theme 3: other symptoms, though in this circumstance it is more about the ability to cognitively process info. and language, not necessarily whether their motor skills allow this).
- **Anxiety:** Feeling anxious; PwDLB may be prescribed antidepressants, antipsychotics, or benzodiazepines to help with this.
- **Agitation**: Feeling agitated, restless, may also include aggression or behavioral disturbances.
- **Social withdrawal**: Withdrawing from activities, socialization; may become less vocal/talk less.
- **Depression/sadness:** Describes PWDLB feeling sad toward EoL––may be due to approaching EoL or just b/c of the disease and situation like limiting activities they’re able to do. *Cgs. may answer ‘yes’ and expand when asked the interview question “Did they experience anxiety or depression in last three months?” (but can sometimes be hard to tell if they are talking about anxiety or depression or both.)

**Relationships b/w Themes notes/memos:** Properties (symptoms) can (and often do) co-occur together and/or with other themes/properties -- e.g., PwDLB may have anxiety after hallucinations/delirium; or may have agitation/behavioral disturbances during hallucinations.

| **Properties** | **Representative/Exemplar Illustrations - Data Excerpts** | **N** |
| --- | --- | --- |
| Hallucinations/ delirium | Interviewer All right, so tell me about his -- you and your husband's experience when he was near the end of life.  Interviewee: He was getting more difficult to deal with his anxiety and paranoia. They were getting much worse. he started having his very active dream and he fell out of the bed. [1926-01]  that last month, there was a lot of, of confusion, delirium, [1927-25]  So like I said, she previously had pretty commonly had hallucinations of cats and dogs and sometimes babies, uh, which she enjoyed, you know. And uh found them cute and comforting, I think. And so, the week before she died, about eight days before she died, she started expressing seeing a man in her room. And I was there and she said, "Do you see him? Do you see him?" And I said, 'No, Mom, there's nothing there, I promise you." [1927-12]  “We had three years of major delusions and paranoia, and there were three and four of … there were times that we went through that he didn't think he was at home. I had to take him out and drive him back in again.” [1923-06]  “She had a cat all this time. I mean, she's had that cat. She was thinking he was a baby.” [1927-17] | **9** |
| Cognitive decline, fluctuations | But we could have a really wonderful conversation about our love for each other and past lives and I can't wait to be with you again. And then he would stand up and I'd say what's wrong? And he'd say I have to pee, and I'd say okay. And he looked at me and he'd say, where's the bathroom? And we had just been having this amazing deep conversation and then once he stood up and everything disappeared and he didn't know where he was. [1927-44]  The other thing that was different [the day before he died] was, he said to me, “your face looks familiar, who are you?” And I said, “I'm your wife [interviewee name],” and he said, “oh okay.” And the same thing my daughter, he asked my daughter who she was, and she told him. and then that night when we came to bed…before he went to sleep, he said, “I recognize your face. But who are you again?” [I said] “I'm your wife, [interviewee name].” And he said “Oh I'm glad. I hope we have many happy years together. And he held my hand, and I said, “I hope so too.” [1926-01]  “He could watch TV, but I think in the end…he would confuse toward the end. He would be upset, and I'd say, ‘What's the matter?’ And [he’d say], ‘Well, this kid's been kidnapped.’ And I'm like, ‘What kid's been kidnapped?’ And he's watching, you know, something on TV, and I'm like, ‘But, Honey, that's TV’ -- I'd say, ‘that's on gun smoke, that's Matt Dillon. It's not real.’ And he'd be like, ‘Oh, oh, yeah.’” [1923-06]  “It wasn't that he was not physically able to get out of the chair, most of what happened with the chair was that he didn't remember how to get out of the chair. … When they talk about things coming and going, like confusion and things like that, he would be so normal that you wouldn't think there was anything wrong with him. And then-- and then you have trouble with real basic things. He never forgot who people were and what was going on. He could tell you about my-- I have a sister that has foster children, and he could tell you all about them and where they came from, but yet, like I said, he could forget how to get out of the chair.” [1923-06]  “Well, the Lewy body was all about him processing information. It got harder for him to communicate.” [1925-06]  “I noticed she didn't understand me the way she understood me prior. She wouldn't, uh, simple [things] like, are you going to bed? She didn’t understand it. The English, it's like she lost it.” [1927-17]  “His physical being, I felt like…his mind was gone several months before his physical self. So, when he really started going downhill physically, not being able to bear his weight, being incontinent, needing help holding a utensil -- when those became apparent and got worse, then I felt like we were approaching kind of that last road.” [1927-31]  “There were a couple of incidences where, um, he would--he would just-- It was almost like he went into this zombie mode where-- Well, I thought he was going to fall out of his chair but, his eyes were wide awake… it scared me so bad. I just kept shaking him...and finally—[he] blinked his eyes.” [1924-16] | **7** |
| Anxiety | Before he passed away, he was still having some issues with the delirium, and that anxiousness that that caused. [1927-25]  He would say he's getting ready to go and I'm like, "Go where?" And he said, "Well, to heaven. I hope." So, yeah. That was an awareness that there might have been a bit of anxiety surrounding that at times. [1927-10]  “His anxiety was-- it was more related to if I was out of his presence--And he was on Clozapine, he was on Sertraline, and all those worked very-- he-- [de-identified] did an excellent job of fixing medicine to keep him as good as he could be at home … But the anxiety at the end was more related to, where was I? [due to separation anxiety] … of course, his sense of timing was not good anymore either. So, a minute to him was a long time … I could be sitting across the room from him, but out of his line of sight, he would drift off to sleep, wake up, and have anxiety because he didn't know where I was.” [1923-06] | **14** |
| Agitation / behavior changes | Well, the last days, he was in the emergency room and he was -- he was really just in terrible shape, um, angry, um, just totally out of control, uh, both physically and mentally. Um, and you know, as I said, it was just totally out of character, his violence, his yelling at people, he was punching at people, kicking, and all of that. So I don't -- I -- I think ultimately, I know they gave him morphine at um, hospice, that I mean, they were trying to sedate him, calm him down. Um, so it was -- it was uh, an agonizing uh, demise. [1926-06]  He would start ripping them [his depends] into little pieces and ripping his t shirts and he was frustrated, and he didn't really know what was going on. [1927-44]  Well, through the whole illness, he was on clonazepam and we couldn't put him on an antidepressant because it made him manic. it made him-- it really brought out nastiness. And so we just couldn't use them. [1927-21]  “She had a cat all this time. I mean, she's had that cat. She was thinking he was a baby. But she was very mean to that cat that last, the month before, she was mean, I had to take the cat with me. She was so mean to him. I don't know why, but she was very mean. Never has she been mean [before]. [1927-17]  “He became violent, destructive. And so it was like he died before he died and that is excruciating. So, if anyone is going through that, especially if the person that they have has dementia, if the person is violent and he has not been violent before, it just crushes you. So, it crushes your spirit to see that because that's not the person that you knew before.” [1927-40]  “At night, I would move, he was in the hospital bed and I was in a twin bed, so I would lower the hospital bed to get next to him. And if he seemed really agitated, I would just lie right next to him inches away and put my arm over his chest or hold his hand through the night.” [1927-59] | **8** |
| Social withdrawal | Last few weeks. He actually, he had gotten to the point where he-- he when he got the illness became extremely withdrawn from the beginning that you know, I would listen to other people and they talked about their husbands doing things and talk. He became very nonverbal. And in the last few weeks. Um So that, but there was a change in the last specifically week, it seemed like overnight he became even more withdrawn and it seemed hard to believe because he was already withdrawn, but we just knew there was something really off. But even leading up to that he was getting where he would eat, but he would immediately withdraw. He, his-- his mannerisms for that net respect. It seems like everything like that changed him. He just seemed like he was not participating in anything. He just wanted to lay down in bed all the time, all the time. [1927-04]  My sister and I did take him out of the facility for a little bit and just tried to enjoy some time with him, but it was really difficult. He just had no spark, hardly any life in him. So we were glad to be able to be with him, but it was not our dad anymore at that point. [1927-02]  “But he was still fairly relational, he always knew me, always knew when I came. And one of his favorite things was singing, so we would go to the entertainment every day that they had there [at the nursing home] and he would sing along. And I noticed maybe the last week or so that he wasn't singing and that was his favorite thing… So, she [nurse] brought it [milkshake] to him and there was also an entertainment that day and he wasn't singing, he barely drank out of that [his milkshake] and she was very alarmed, she thought, you know, this is not like him. So, that was our first real scare.” [1927-07] | **4** |
| Depression/feeling sad | “Well, I think he did, between me and the help that I had, we knew he was sad. I knew he was aware of how- I don't know if he was aware of how close he was to death, but he was aware that he was dying. And he hated that, [laughs] he hated not being able to talk about it because his word searching was terrible.” [1924-10]  Interviewer: During the last month of [de-identified] life, did she have any feelings of anxiety or sadness?  Interviewee” I mean, she didn't say anything like, ‘oh, why is this happening to me? It's not fair,’ or anything like that…she was mostly placid during the last month, but there are occasions where I put on a song as we were strolling around outside, and she would smile or something. [1927-22]  “Sometimes she would be very sad and just cry and I didn't know why exactly.” [1927-68]  “Well, through the whole illness, he was on clonazepam and we couldn't put him on an antidepressant, because it made him manic. Like it made him---it really brought out nastiness.” [1927-21] | 8 |

1. **THEME LABEL: OTHER SYMPTOMS (N = 28)**

**Thematic properties:**

- **Excessive sleepiness:** increase in sleep or desire to sleep. In some cases, may be asleep for pretty much the entire day.
- **Trouble breathing:** Difficulty breathing on one’s own; may have an oxygen machine or something else assisting them with breathing or not; labored breathing toward very near-end.
- **Loss of appetite and/or difficulty eating**: PwDLB loss of appetite/not wanting to eat; or complete inability to feed or help feed themselves; may have trouble taking in food or holding it down. *Different from ‘eating/feeding’ under theme 1: motor, which PwDLB still has appetite but has trouble feeding themselves e.g., difficulty holding a fork.

| **Properties** | **Representative/Exemplar Illustrations - Data Excerpts** | **N** |
| --- | --- | --- |
| Excessive sleepiness | That week before, he was extraordinarily sleepy, which is really saying something because one of the symptoms that I talked about in the past [is] he slept a lot. He's always slept a lot, but that last week just slept so [much]. very difficult to get him to even take a pill and then he was gone [asleep] again. [1927-23]  A week before he died…[he] was really having a lot of more mobility issues and just being lethargic. [1927-67]  He was sleeping all day, it seemed like. He would just be asleep in his wheelchair out in the common area. [1927-02]  He slept almost constantly, and when he wasn't asleep, we did everything in our power to help him back to sleep. [1927-21]  “You could see that he was declining. He still slept quite a bit during the day, but he didn't get up when -- when all this started he would get up and walk and wander in the middle of the night and he didn't do that. Now he slept all night when he went to bed when I put him to bed he wasn't resistant.” [1925-09]  “It got to the point in the last couple of months where he had so little energy and he wouldn't have his eyes open, but he would eat if I fed him… so you would think he was sleeping, but he was awake and wouldn't respond.” [1927-11]  “Really the last month he was pretty much just sleeping, and he would occasionally sort of wake up, but he was pretty much out of it by then.” [1927-60] | **12** |
| Trouble breathing | The whole week, he started having a lot of trouble. It sounded like he was having trouble breathing. It became very, especially the last couple of days over the weekendHe was doing very rapid, somewhat noisy breathing. [1927-23]  During the last couple of days, like I said, she had labor breathing. those last couple of days were very trying, with her laboring to breathe. And, of course, the last day, we got to the point where we were injecting the morphine orally in the corner of her mouth every 2-3 hours because of the labor intensity of her breathing. [1923-12]  “Near the end, he was breathing pretty fast, according to my son, and so they would give him whatever medication they gave him and that would slow down his breathing a little bit more.” [1927-07]  “She would breathe through her nose and her mouth would be closed. In the end, mouth was open, and she was breathing through her mouth… It was just, her body was diminishing. She wasn't able to keep her mouth closed and breathe through her nose. The jaw was down and her mouth was slipped, and she wasn't controlling anything.” [1927-22]  “The last, like a week before he died, he was taking like, two breaths every minute, but then he kind of recovered and started breathing-at a more normal pace. [1927-60] | **16** |
| Loss of appetite and/or difficulty eating, weight loss | We backed off of a lot of things. We backed off trying to feed him. He clearly had lost his appetite, backed off trying to get him to the bathroom and you know, did sponge baths as opposed to taking him into the bathroom. So the only thing we really tried to do was to continue to give him medicine. [1927-23]  He was so thin, he was eating very very little at the end and we were giving him ensure everything to keep him…strong enough. [1924-04]  He at towards the end, the symptoms just got really hard for everybody and I think quick decline were his lack of food, like he stopped eating and, of course, he was drinking Ensures and did very well with that, but he had lost so much weight. [1927-02]  “I had a hard time getting him to drink water, a very difficult time getting him to drink water. And I tried everything, sippy cups, straws, spoon, pools of water. It was, he just, he was just shutting down. Somehow in my tenacity I just thought that I could just push it through, because at the time I wasn't noticing these things as elements of his decline. The weight loss, that was disturbing.” [1927-47]  “Definitely the weight loss [was a sign]. How he was eating, it was so minimal. And I read something how ice cream, people with dementia really enjoy ice cream, because it kind of brings back, you know, good memories and it's tasty. And so, we did start to give him ice cream when he would refuse soup or any kind of typical food.” [1927-59]  “Within the last week, 10 days, maybe, she just stopped eating. They said we can't feed her because most of, she'll swallow the stuff in her windpipe, and in the end, so she didn't get anything. And then, so I said, ok, well, this isn't gonna take very long. And so she just got weaker, and weaker, and weaker… It was the beginning of the final stage.” [1927-22]  “He did say the day that he died that his stomach hurt him… he pointed, like he didn't say it, you know, [he] couldn't articulate. But I could see in his face and I was asking him ‘did something hurt you?’. And he pointed to his stomach.” [1927-53]  “When he stopped eating, I was really, really concerned because I couldn't tell whether he had gotten to the point where he couldn't eat or because he didn't want to eat.” [1926-02] | **11** |

**End of Life Trajectories**

| Death unexpected | “I just didn't expect it had to happen so quickly because he was walking and talking, then the next week he wasn't able to really do anything. … But I don't think he expected it to go that quick either, because he did say this is happening so fast and then the next day he wasn't even talking. So, he knew…he just didn’t expect it to be so fast.” [1923-01]  “That afternoon, they found him a place at hospice house. He went from in the morning, not qualifying, to [going] to Hospice house that afternoon. So, I guess that was the euphoria that they talk about, maybe. They had the afternoon that he was telling jokes. He went through-- at hospice, his respirations, I think he was down to, like, 12 two or three times, and he’d just come right back up.” [1923-06]  “I found it shocking and painful that two weeks before his death, he was walking around, and we were doing things together and I would-- I was feeding him…and we would sit and watch TV together and things like. That seemed kind of like a normal time. And then two weeks later he was done. It still shocks me that [he] went so quickly.” [1925-06]  “It was a shock to me. And of course, it was shock to the family because we didn't––my anticipation of his life was going to be that I would end up having to have in home care … [but] he was still eating everything I gave him. He had many falls and of course the neurologist suggested that, ‘this is what we're going to be going down.’ … and that none of that happened. Just that quick, he died. So, I don't know what he died of. I don't think it was the dementia that caused his death, [or] whether something else happened.” [1926-09] |
| --- | --- |
| *False alarm – fluctuations prior to death | “It's hard to say [if he got the right amount of help for his pain] because we couldn't tell, you know, after a while when he was–– you know what I'm saying? Like I'm thinking of the day that he died. Like beforehand, I think he was okay. I think he was functioning, and he was okay. And the week before he died, that's the time when he would like rally back that I thought, well, maybe he's going to come off hospice. Because he was hungrier. He was drinking more. He was well hydrated. He was more alert and like he -- that week he rallied back and then -- then he passed away.” [1927-53]  “It gets really hard to tell, because sometimes, I mean, people just go up and down with these kinds of issues, with dementia and all that. He could have a bad couple of weeks and then kind of have a good few days. So, that part, I think to me was the unpredictable part. … I guess there was a decline when I kind of piece it together. And I just kind of, maybe just hoped that he was going to rebound, but realistically it was a steady decline [in the last few months].” [1927-100] |

**RESEARCH QUESTION 2 (RQ2):** What factors are associated with positive or negative experiences at the end of life?

1. **THEME LABEL: INFORMATION (N = 24)**

**Thematic properties:**

- - **Information about the disease**: info. On DLB, particularly how it is different than other types of dementias, and common symptoms, stages of the disease, progression, etc.
  - **Information about what to expect at EoL for persons living with LBD**: what caregivers may expect the PLWLBD to experience once close to EoL. *Similar to ‘info. about the disease’ but more focused on the EoL, though for some cgs., these may overlap.
  - **Information on pharmacological or non-pharmacological care management**: Caregiving tips/information, info on pharmacological or non-pharmacological symptom management or lifestyle changes that may help with EoL.

| **Properties** | **Representative/Exemplar Illustrations - Data Excerpts** | **N** |
| --- | --- | --- |
| About the disease/DLB | I think you have to try to learn as much as you can about it. When we first got the diagnosis, I did a lot of reading and participated in some podcasts and things like that to try to get as much information. And I mean, I already had a foot up from most people because of my background but you need to learn as much as you can. [1923-10]  The other thing, and I say this to even people beginning the journey, especially with Lewy body, is read. And I don't mean internet stuff. I mean, read what you can read, the LBDA, ask your doctor. Some doctors need to be educated because they don't know everything, especially primary care… I would say, absolutely educate yourself. [1927-10].  Interviewer: What are some important things that you would like others to know in order to help improve that end of life experience for other people with dementia, with Lewy bodies and their families?  Interviewee: I think that if in collaboration with the doctor and that now the doctor would be the one who diagnoses. So, the neurologist, I would think would have more of this information [like] understanding like the stages that happen, you know, what you expect in -- during those stages… Now everybody meets those stages differently… I tried to understand but I think already from my dad, it was -- we didn't catch it in time, you know what I'm saying? But for other people, understanding those stages, understanding what to expect [from] hospice… just understanding what may happen, you know. Giving you the heads up,’ this is what happens.’ [1927-53] | 9 |
| What to expect at EoL | Interviewer: Are there any things you would do differently around the circumstances of [patient]'s passing, or anything that led up to it?  Interviewee: Just more information. I wish that I had knew more about kind of what to expect…I don't know if all the patients go into delirium like that. I don't know. I just wished that I had been better prepared to have recognized what was going on. Even hospice didn't know. [1925-25]  Interviewer: Did any of the doctors or other health care professionals discuss with you what to expect at the end of life and people with dementia with Lewy bodies?  Interviewee: I guess I have to say yes on occasion. The reason I have a hard time with that is because…I have gone through this with my mother several years before… I had also educated myself especially with my mom and then, especially with [patient], I had done a lot of preparation in terms of reading specifically what kind of things could happen. So they did offer a little bit, especially as the end got near… For me, they tended not to be theoretical questions. Don't tell me this may happen... Once it happens -- That's what [information] I was after. [1927-23]  So when I had read about -- well, it was Dr. [de-identified]'s article, the only information I could find about what to expect at the end. And when I read about people's experiences I was terrified and anticipating the difficulty swallowing and a long period of immobility. [1927-01]  “I guess the first one [thing I would tell another caregiver] would be that if the person has suffered a lot––…[with] Lewy body dementia, I don't know, if they suffer a lot––But if, if they do, then I would hope that the caretakers would be told that, that can happen. And to get as much support as you can, from family and other people.” [1927-40]  “Hospice never told me that he wouldn't talk to me or anything like that again because I guess they probably don't know for sure. But it would have-- it might have been more helpful-- like, I kind of felt like that from the beginning, but it might have been more helpful to his daughters if they had said it to the daughters.” [1923-06] | 14 |
| Caregiving-specific information | I did a lot of reading online. Not that everything online is real or true. But if you find that nine things out of 10 are the same, that it's like – okay, that's some good probability. And I really helped myself by looking around and seeing what was going on and say, ''Okay what can I do to make it better?'' And I would research things. I found a company that makes shirts that look like they're button-down…but they're Velcro… I found him some shoes that have Velcro as well…there's little improvements that go a long way and meet him where he's at. [1927-44].  One thing I would say is to be educated about Lewy body and be aware of side effects of medicines. And for people to understand, especially women caregivers because most of them are, you don't have to say “okay” to what some professional or non-professional says to do, whether it's a medical treatment or whatever it happens to be, you don't have to say yes. It's just a matter of asking a lot of questions. It's a matter of pushing back and saying, “no, that's not appropriate for me or for my partner.” [1927-23].  I was reading in one of the articles about the burden on caregivers for LBD. One element of that burden being the sense of being not fully competent. And I was thinking -- how would it ever be possible to feel competent when you're walking into a room and this person that you love is saying, “I can't find my fingers.” [1927-01]  “I have gotten a lot of support. I told you that I had a support group for people whose have dementia. most of those have Alzheimer's, some frontal lobe dementia, but nobody really had lewy bodies… so I had that group which is invaluable to me to have a group of women that you could say stuff to, and they got it. They understood and also, they were an incredible resource for material. You know, I got two wheelchairs from, I got two walkers, different walkers, and just information about, ‘well, what kind of diapers did you use?…And how did you deal with this?’... just tips on how to deal with the many things.” [1927-75]  “Well, I was doing videos for a long time, like a minute and a half videos telling people what to look for and how to do this and how to do that with people that have Lewy body dementia and Parkinson's. When I see people, I tell them, you know, make sure that you get the ergonomically correct silverware. The thing that I wish I would have done sooner and I try to tell other people to do sooner is when--make sure that you get the Parkinson's walker. Because you have to teach him how to use the walker. And if the Lewy body is too far advance, you can't teach him how to use the walker. So, you know, something that you have to do early in the disease so that they can benefit from all these apparatuses that they have, [like] their ergonomic silverware. I also, talked about these plates--they're called dipping bowls and they're the size of a plate but they've got a ridge on them and it's the best thing for people that are having a hard time eating and controlling their food…and the size of straws make a difference and the type of cups that you buy.” [1924-16] | 7 |

1. **THEME LABEL: SUPPORT (N = 30)**

**Theme description/definition**:

- - Sources of emotional/social support include family (children, siblings, etc.), friends and neighbors, support groups, and even clinicians and hospice workers.
  - TYPES of support & supportive activities described by caregivers: empathy & expressing concern, acts such as preparing food for the family, spending time with the family, being available to the caregiver, showing support through formal groups (i.e., support groups).

**Thematic properties:**

*Things caregivers did:*

- - **Attending support groups:** In-person or virtual (could be synchronous or asynchronous) support groups for caregivers and/or caregivers of PwDLB; (in some cases cgs. attend individual counseling or therapy).
  - **Fostering relationships/having a sense of community:** Cg and/or PwDLB’s fostering their relationships and community toward EoL, e.g., religious community (or other organizations they were involved in), local community/neighbors, former colleagues, friends and extended family, etc.
  - **Receiving** (formally “accepting”) **help from others:** Both *asking* for help from others (includes formal or informal caregivers) and ability to *accept* help from others. In some cases, cgs. may have wanted to have been offered help from others (e.g., family/extended family/relatives) but weren’t. Common thread: you can’t do it alone.

*Things others did that helped caregivers feel supported:*

- - **Spending time with the person living with LBD and/or caregiver towards the PwDLB’s EoL**: Loved ones (friends, family, colleagues, peers, etc.) spending time with the PwDLB as their end-of-life approaches. They may reminisce on old stories, tell jokes, etc., or just silently spend time next to them.
  - **Showing support for the PwDLB and caregiver**: Various ways that others can show support for the PwDLB and caregiver (outside of the ways mentioned above/in other properties). (Coding note: collapsed/combined former properties ‘Being empathic and expressing concern’ / ‘Being available to the caregiver’).

| **Properties** | **Representative/Exemplar Illustrations - Data Excerpts** | **N** |
| --- | --- | --- |
| Attending support groups | I think support groups are really good because lots of times you learn thingsIt gives you some insight. I think a little bit different with the support because people are really creative [with] the way they find to get through things and to handle things. [1923-10]  There were three others [spouses] in a larger [support] group. And we used to go to lunch when we could and coincidentally, all the husbands had Parkinson’s, but they also had Lewy body. And so, this is when [patient] was still home, I would say, "Oh, I'm having lunch tomorrow with the dementia girls." [Laughs] The dementia girls. But, yeah, you have to embrace it. you have those people around you for support. [1927-10]  Well, I think going to support meetings for the caregiver and also for the patient when they're first diagnosed is very good. After a while, they can't really keep up with this…but the caregiver should continue the support group because it helps to vent some of your frustration. I didn't do that. I vented with my therapist…But I think the support group is good because you hear the experiences of other people and then you don't feel as alone because you find out that there are similarities and when you find those similarities it eases the burden. The same with this grief support, even though our husbands died from different things and at different times I have found that we have similarities and that I'm not going crazy and I'm not the only one that's angry or I'm not the only one that cries a lot. [1926-01]  I think the support group is really important, to reach out and find other people who are going through similar things. That was invaluable to me and to [my husband]. I think just so that you don't feel so alone in dealing with it. Realizing that you're going to play an educational role to other people. Like, in my case, I had to educate the caregivers about some of this. [1927-12]  The other thing I did, which I really appreciated, through elder Network again was just took some little mini courses on art, some doodling, and some drawing, to try to keep in touch with the creative side of ourselves so that we don't feel totally stuck. And I was able to do that through elder network and the caregiving groups really provided opportunities that all I had to do was take advantage of. So, I would encourage another person going through this to take advantage of the support. [1925-07]  I wish we had joined the caregiver support group because I'm in a support group right now for a medical issue I'm dealing with and it's so clear to me that you get great information from fellow patients who caregivers who've already gone further down the path than you are and all those groups are facilitated by people who also have expertise, you know, clinical expertise or counseling expertise. [1927-67]  “I learned more about lewy body from being on the Facebook support groups for lewy body caregivers. I learned more about it there than any place else. None of the associations or medical world can tell you the day to day living experiences because they're so varied. And knowing and reading about that helped to prepare me. So I would say get on one of those support group. And it's scary because of the horrible things people go through, and some of them go through it for 10 years. I didn't know how long it was going to last, and I didn't know what was going to be next, and that's scary. But it also helped to prepare me so that when some of those things did happen, I was thinking, ‘okay, this is the [de-identify] body and here's what I need to do’.” [1927-77]  “I never did any of the chat lines and things like that-- one time I think I called Lewy Body Association or something, and it was after hours and they're like, ‘Oh, yeah, leave your name and number,’ and I'm like, you got to be kidding me? But you need somebody that you can, you know-- and you will need somebody you can vent with-- because it's enough to drive you crazy.” [1923-06] | 13 |
| Fostering relationships & a sense of community | Well, I think it's really important to nurture the relationships you have with the people that you have, [so that] you have a support system. I think that's really important… A lot of people I know don't have very many friends or aren't very involved with some people. It's a small circle of people or some people don't get along with their neighbors. They don't go out with -- they don't, they're somewhat isolated, and I think you have to be as involved with as many people as you can. [1923-10]  Interviewer: Well, how about emotionally, how have you been? Are you getting support?  Interviewee: Well, I haven't -- my son lives in [de-identified], so I mean, he's wonderful, but he's far away. I have really no family or friends close by. [1926-06]  “it's hard to ask for help because you don't know how long this is going to go on and you don't want to burden your friends and family. And I know that my daughter and my daughter in law both agreed that they were going to take it upon themselves––they were very helpful, I have a good family. But they knew they couldn't be here all the time. But they were planning to take it on themselves to ask like people at my church to let my pastor know that I needed help. So it wouldn't be me asking, it would be them asking for me, which would make it a little more comfortable. But people are afraid to offer.” [1927-77]  “Probably the biggest advice is, you need to surround yourself with friends and family because you can't go through it alone. You have to have your support––people you can just vent to, people that care for you and what you're going through… those connections and relationships are so important. And if you don't have that…I can see where someone can fall apart because the problem is bigger than one person and it's important to have that base support group for yourself to get you through it.” [1927-03] | 9 |
| Receiving help from others (*both asking and accepting*) | I think an important thing to do, which I had trouble with at times, was accepting help. People kept saying, "Can I do this? Can I do that?" And I said, " No, no, I got it covered" And you should take as much help as you got offered. People want to help you and I was just used to [taking] care of everything. We didn't need anybody doing things. And then we did, and it reached a point where we really needed it, and it was needed for him so I could do the things I was doing and so that he could get care and attention that he needed. [1923-10]  Have your family around [to] get support…You can't do it by yourself…Have family support or [if] not, get friends, but don't do it alone. [1924-4]  One of the problems was, I got it in my head that nobody else could take care of him, I can take care of it. That's not true. [1926-01]  Asking for help, that's important. And that's something it took me a little while to do. I couldn't ask friends to help. But towards the end, I did… So anyway, ask for help. [1927-35]  “I think it's hard to say what they [people/other caregivers] should know because things happen so unexpectedly, it's such an up and down experience. And you don't know what each day is going to be like, some days are better and some days are worse. But I would want people to make sure that they…try to have help. Because there are times when the caregiver cannot have any sleep or very little sleep because the person with lewy body is up and down day and night. You're going to be up and down with them because they're doing things and they're falling and they're hallucinating and…you can't rest easy even if you have time to rest.” [1927-77]  “I think getting outside support is critical. Absolutely critical. It certainly was for me. I couldn't have done it without the adult daycare that gave me time to do some errands and go to the grocery store and sometimes I would just sit, sometimes all I could do was sit out here on the deck, drink some coffee.” [1927-75] | 14 |
| **Things others did to be supportive:** | | |
| Spending time with the PwLBD and/or cg. towards EoL | Well, I think we had gotten into a really good routine with the people I had coming to help me. I mean, we really had it down to a system.We had the girls, our daughters, and their spouses were coming, alternating time, had friends coming in, and then I had professional help through the home health agency. [1923-10]  As it turned out, my closest friend -- my college roommate, flew up here to be with me. And I don't know how I would have done it by myself. So, I would say even if you feel that you've had to be competent and self-reliant through most of this experience, if there's someone who can be there at your side at the end and in the days that follow, make that happen. [1927-01]  The only thing I would do differently is the thing I didn't have any control over and that was having my kids at home with us that--those final two weeks. I really wanted them to come. [1927-21]  “He got a lot of love. There's no question about that. I mean, the family rallied. In the last week of his life, the family was there with him all the time…We were there pretty much all of his waking time.” [1925-06]  Interviewer: Did he get any help in dealing with his feelings of anxiety or sadness?  Interviewee: I think just people being, trying to be supportive. Just conversational. He had a social worker [that] was visiting every other week, I think. And then of course his nurses talked to him a lot. [1927-100]  “About two weeks before his passing, he had visits from some friends. We had incredible support from his friends. … Some of his friends came to visit him every few months. Can you imagine you going to another city to visit a sick friend several times a year? So humbled and proud of what good friends they were. Anyway, that visit was a good visit. [de-identified], he wasn't very talkative, but he was clearly and listen to everybody and reacted.” [1925-06] | 9 |
| Showing support | I would ask for a replica of my experience in terms of a strong support from the hospital community, not only the physicians, but the nurse practitioners and physician's assistant down to even the people that came in and cleaned the room, just the whole support, understanding that we needed that love and caring. Also, the nurse practitioner who followed up from neurology every three months. That continuity of support was very valuable. [1925-07]  The overall experience was excellent. I can only applaud the [name] Hospice Group, from the pastor right on down to the relief nurses over the weekends because they were genuinely concerned. They expressed their concern of the condition, they offered their schooling to the situation and, of course, in that kind of profession, you're used to those type of situations and know how to deal with them from a personal standpoint, being strong and being supportive to the family member. And I just applaud them. I mean, they were excellent. Even now, I get cards from them, I get calls from them. They want to know how I'm doing, is there anything they can do for me. [1923-12]  I had so much support. I just, and I think I've said it so many times. Everybody's probably sick of hearing it. I couldn't get over how much support I had. I had too much food in the house.… My one neighbor kept bringing stuff [food] and everybody kept saying, "You got to eat, you're going to lose weight.” I actually put on a few pounds, and it was because I was constantly having to eat these nice meals that people were bringing me. [1923-10]  Every day there was somebody there [from hospice], the nurse or a chaplain or -- I had somebody else I think for emotional support, but they were great…. Hospice, they were terrific. [1927-54]  “They have been roller coaster emotions. However, again, I've had very supportive family and friends and even medical support workers, even being a part of this project is part of my healing…I would ask for a replica of my experience in terms of a strong support from the hospital community, not only the physicians, but the nurse practitioners and physician's assistant down to even the people that came in and cleaned the room, just the whole support, understanding that we needed that love and caring.” [1925-07]  “Surround yourself with people that are going to be a support group for you, that can help you out in these difficult times. Good doctors that also can do that, can help you to maneuver through this.” [1927-25]  ***Being available (i.e., just knowing others are there for them)***  They [my daughters] were here in the middle of the night and one daughter stayed with me and the others even came in the middle of the night if we called them. [1924-4]  I did not expect to have as much help and support. I did not expect my daughters and their spouses to put so many things on hold to be there. [1923-10] | 6 |

**3. THEME LABEL: HOSPICE (N = 32)**

**Theme description/definition**: *Utilizing hospice/home healthcare services*

**Thematic properties:**

*Factors that help make it a positive or negative experience (properties/subthemes):*

- ***Communication* with hospice healthcare workers**: (both frequency and quality of communication): more communication/empathic communication can make experience more positive, e.g., using empathic comm., building rapport).
- ***Care coordination***: between caregivers, hospice workers, and/or healthcare workers at a health facility. Can increase cg. burden if care isn’t coordinated effectively.
- ***Quality of care***: How well the cg. feels the patient was cared for by hospice at EoL (including home healthcare equipment/care environment, pain management/medications)
- ***Caregiver education****:* recommendations, caregiving tips, etc.
- ***When to start hospice***: Starting hospice at the appropriate time, e.g., too early or too late can make experience positive/negative.

*^How this impacts EoL experiences (discussion):*

- - - Can increase or decrease *caregiver burden.*
    - Can provide *caregiver education.*
    - Can provide *quality EoL experience* for PwLBD (e.g., chaplains, social workers or volunteers)
      - Recommendations on equipment can reduce cg. burden/more positive experience.
      - Properly managing pain = more positive exp. Not properly managing pain or over-medicating PwLBD = negative.
    - Starting hospice early isn’t necessarily bad but more hurdles/issues with insurance.

| **Properties** | **Representative/Exemplar Illustrations - Data Excerpts** | **N** |
| --- | --- | --- |
| *Communication* with hospice nurses/staff  (frequency & quality) | Both the Hospice team and the nursing home team were careful to check with us about increasing and to explain the reasons that they were recommending increasing doses. [1927-01]  Our experience with hospice was excellent. [They’re] courageous people who are willing to come into your home and your life and your family at the worst possible moment of it. We did have one caregiver who showed up who just freaked (patient) out and I told the nurse about her and she never came back. And the nurse recruited a caregiver specifically that she knew would be a good match and the two of them were amazing. The chaplain was great. He had a really heavy caseload, and he only came three times, but he really talked to (patient) very frankly in those first couple of months before he really couldn't talk anymore, and I think helped him. [1927-21]  The overall experience was excellent. I can only applaud the [name] Hospice Group, from the pastor right on down to the relief nurses over the weekends because they were genuinely concerned. They expressed their concern of the condition, they offered their schooling to the situation and, of course, in that kind of profession, you're used to those type of situations and know how to deal with them from a personal standpoint, being strong and being supportive to the family member. And I just applaud them. I mean, they were excellent. Even now, I get cards from them, I get calls from them. They want to know how I'm doing, is there anything they can do for me. [1923-12]  “And I called my home health aide, [de-identified]…, we stayed very good friends, and to tell her that she didn't have to come in, this was about 6:30 she was coming at 7:00. And she had always said to me, if he passes, call me immediately even if it's the middle of the night, I want to come out. And she sat, she came right over and she sat with him… from seven o'clock until my daughter's arrived from [de-identified] at 11:00.” [1927-59]  “Their staff was really good at-- because they would come usually during the day and then I'd get there at 4:30 every day. And usually, they'd already been there and done like showers or whatever on the nurses visits. But they always followed up with emails. So, they kept me informed. And then, especially in that last week, in particular, I saw the nurse every day. And we would confer about his condition and decide what to do on a daily basis. How to respond to what was happening.” [1927-100] | 9 |
| *Care coordination* | I had to go out and ask the nurse at the nurse's station on that Friday night. He had been there four nights at that point, and this is less than three days before he died. I said, ‘Do you have a hospice?’ And they're like, ‘Yes, we do.’ And I'm like, well that's what we want, and they're like, okay, so then we had to call back the other place [to] get the possible bed picked up. It was a major hospital that we were at. It was, I think, very typical in that there should have been a more coordinated effort to talk about hospice pretty early on and they didn't. [1927-67]  “Yeah, the nurses in a way were more of a nuisance because of the constant changing times and days and, you know, showing up later because I had my own home health aide who was on a regular schedule three times a day and then I'd have to call her and tell her not to come, because they were just here. … The home health aid was excellent, the social worker was beyond excellent, and the nursing, I wouldn't give a good grade.” [1927-59]  “I really thought they were, they [hospice] were very helpful…because they could check up on him more than the staff. So that extra care and the clear, scheduled visits. And so, it was more predictable for me to know when he was getting services.” [1927-100]  “They had a lot of turnover in the beginning in terms of the hospice nurses and there was one nurse who couldn't keep up with the paperwork for hospice.”[1927-60] | 10 |
| *Pt. quality of care*  (Pain management/ medications, providing home healthcare equipment) | “For the first couple of months after he was started on hospice, I was impressed that both his quality of life and my quality of life seemed better. I got more help in the home at that point and more of the equipment that would be helpful.” [1924-10]  “The care that he was given at the hospice was just amazing. I think it couldn't have gotten any better in this situation. I feel that ⁠we were⁠ on top of it, when-- just throughout the whole the care giving experience, I was able to solve anything that would come up and figure it out. I feel that everything went well even at the end.” [1927-03]  “Overall, [hospice experience was] positive. They did a great job, I think. They were very attentive to, to [de-identified] needs, what they really would be.” [1927-51]  “Hospice was wonderful…The Hospital called Hospice, and they had me call, not an ambulance, but having called the fire department, moved him to his bed and then they brought in a hospital bed. Everything came very quickly. The setup was really fast, and then we put him in the bed and he was in the bed from then on for the for the next six days until he passed away.” [1923-01]  “And they [hospice] were good, the medication came fast, we started on a really good routine. They were there every day, they called. But they were wonderful. I felt very much supported by them.” [1923-01]  “I just thought both [de-identified] and [de-identified] were so attuned to dignity and respect and individualizing and I just never had any doubts about [de-identified] being treated well. I mean, and that was just such a comfort and relief.” [1927-31]  “But so after getting him into that chair, I never could get out again by myself. That's when the hospice evaluator came and was like, okay, clearly he is ready for hospice. And I said I really need a hospital bed, just really need a hospital bed….a nurse came in the morning…she came late and I was-- I kept calling because he was sitting in a pool of-- just soaking wet, his shirt was sopping wet. The mattress, it was so long. She wasn't supposed to come at, I can't remember, 9:00 or something, and it was like 11:30 he was-- everything was wet, it was wet through the mattress pad. I mean, the mattress pad waterproof, so-- and he was sitting in this pee and I couldn't do anything about it. I couldn't move him. And it was pretty awful actually. I understand she had other patients and all that stuff but [still].” [1927-75] | 23 |
| Starting hospice at the *appropriate time* | Interviewer: Do you feel that you were prepared for what to expect at the end of his life?  Interviewee: I think if we had been able to have the hospice and everything, I would have been prepared.  Interviewer: Okay, because you hadn't gotten it that far [started hospice?]  Interviewee: Yeah, because I think that there would have been enough discussion and prepared me at least psychologically, you can never be prepared emotionally at least psychologically. I would have been prepared. [1926-01]  From the from the perspective of Hospice it was way too early. They didn't want to spend any money on him because they we took two years of their money. And that was very obvious, especially as time went on. Um It was very obviously it was very helpful to me because we ran into many, you know, complications with a urinary tract infection with um So bed sores, you know, things that came up, He lost the toenail, things that came along that I could have. I had a nurse that I could talk to. [1927-23]  “I contacted hospice a year before he passed away, more than a year, and they're like, ‘Well, we would love to help you, but he doesn't qualify.’ And the help that was available to me in the last two or three months that he was still at home was for somebody to come here a couple of times a week. And I'm like, I don't need-- that's not what I need.”[1923-06]  “Had we call hospice a week before, it would have been better…I don't know, I mean, but I've heard from other people, don't wait too long to call hospice, and I thought he was calling them in enough time but it took--they have to do the intake, I understand and then they assign a nurse and the nurse they assigned happened to be on vacation, so, they were gonna wait for the weekend. So, apparently whoever did the intake did not recognize that it was urgent either. And so it's kinda just like a mess.” [1927-07] | 9 |
| *Caregiver burden** | My girls never wanted it to be where I lost myself in the process. I think that's one of the reasons they kind of were pushing for the Hospice because they could see that, especially with COVID, I was it was becoming overwhelming, because…I didn't feel comfortable leaving him alone, I guess. So I just was isolating even more. [1924-4]  [Don’t] be afraid to use hospice when the time seems right because they do kind of help everybody. Mainly the patient, the person with the disease, but the whole family gets assistance, support. [19xx-xx]  I would have sought for help, definitely more help…Even though I had help, I had two caregivers who came four days a week, they were just for four hours a day. And I think I should have gotten help for the nighttime so that I could sleep… I think as caregivers, we forget that we have to take care of ourselves or there is no one to do the caring. [1926-01] | 3 |
| *Caregiver education** | I'm glad we signed on with hospice and I felt like we had a lot more support that way. We could call anytime day or night and ask questions or say, "Hey, we're observing this." [1927-12]  I had two different nurses, and very good about going over side effects and what to watch for and when to give more and so forth and it was all the liquid stuff that was easy to handle. And at first, he was wearing regular undergarments and then he went to using Depends type things [so] they had some suggestions on that, and it made it work.” [1923-10]  My third hospice experience, I think that I had answers to my questions when I raised them. I had people checking in with me who could really speak specifically to the things I was seeing and choices we had made ahead of time. [1927-21]  “[Hospice experience was] very positive. Once I got involved, they were close by, they were patient, they were kind, they did a lot of explanation and a lot more explaining of what was going on with his body and some of the things that the doctors didn't tell us about that, you know, just things about how organs are-- organs start to get tired or lose their nourishment or whatever. So I think they gave us a little bit more information.” [1925-06] | 5 |

**HEALTH FACILITIES for hospice (staying at home vs. healthcare facility during hospice care) (N = 11)**

**Properties:**

- ***Quality of care***: patient-centered care (e.g., good care/nurses/care team, focused on pt. and managing QoL), enough attention paid to pt., good nurses.
- ***Care coordination*** between informal cg/family members, HCF workers, and hospice workers
- ***Health facility environment*** [coordination between staff and/or staff and family members, family-friendly environment, low number of patients (or too high/understaffed)]
- ***COVID-issues*** (limited access)

***Helpful when***

- Family-friendly environment
- Good nurses
- Low number of patients

***Unhelpful when***

- Poor quality of care/not enough attention paid to patient by staff/understaffed
- Coordination issues between care facility staff & hospice nurses
- COVID (limited access to care facility for caregivers)

| **Properties** | **Representative/Exemplar Illustrations - Data Excerpts** | **N** |
| --- | --- | --- |
| Quality of patient care | I felt a little bit, not in the dark, but I guess, less than underwhelmed by the care that they gave, and some of the staff didn't seem to have quite as much compassionI was starting to think that I need to move him somewhere else, but between the hospice service and the staff or the leadership there, they kept assuring me that they were going to do everything to make it great. [1927-02]  I think I was surprised at how little the staff really understood the disease. And how shocked they seemed to be at her behavior, you know. But most people there didn't have Lewy Body and so I think the accelerated aggression and paranoia. Most of the people were pretty passive. I think normally when people think of dementia, [they] assume somebody is going to be kind of withdrawn or in their own worlds or whatever, but my mom was not. She was hard to handle. [1927-12]  “[I would tell others that] if they see that decline coming, involve the doctor…and get Hospice in there as soon as they can if they don't have home health or anything… Anyone try to do it alone—have somebody that will relieve you and give you some time for sanity.” [1927-17]  “What would have made it better would have been to not be in the facility…I think with all of the turnover, that is natural to the environments, that you're gonna have some staff that really is just there for a paycheck. And isn't going to be invested in somebody's family. [1927-41]  “There are a lot of times where I was disappointed…they just, wasn't always enough staff. And so, and I had particular expectations that they would bathe him more.” [1927-100] | 5 |
| Care coordination | Felt like because the facility was so poor in a lot of aspects and handling everything, it felt like we had professionals in there. This is what they did. And they were actually seeing what was going on in the facility. [1927-12]  I felt like he should have some social time but also some rest time, and their response was, "Well, he just needs to stay in his bed the whole time. We can wheel his bed out into the day room." And the hospice nurse felt like that was acceptable, and was like, okay, we could do that, I suppose. That way your staff doesn't have to constantly move him in and out of the chair. Well, turns out the bed couldn't go through the door of his room. So, about a week prior, maybe two weeks prior to his death, he was like basically made to be bed bound, which I felt like wasn't what I had asked for or what I want him. It was a total failure in my opinion because they weren't adjusting him, moving him left to right, and he was getting bored. [1927-02]  “There were the inconsistencies on what mom was allowed to do and it would be based on shift, it would be based on nurse, even though there were written orders that she could help clean. That was, you know, everyone had a special moment to do. My mom likes to clean—She wants her environment clean. And she would get yelled at on one shift and then told the next shift it was okay. It should have been okay. We signed releases… that would've changed the world a little bit for my mom just having some consistency.” [1927-41] | 3 |
| Care environment | He would just be asleep in his wheelchair out in the common area so I asked that he be allowed to rest in his bed more during the day, and their response was, "We just don't have the people to be able to move him," because he required at least two people to move him. [1927-02]  “The hospice room had a bed in it and-- or a couch that folded out and [de-identified—PwDLB’s daughter] would sleep on the couch.” [1927-51]  “So, the only thing that, that I imagined being better would be some place like hospice that he would still have privacy. You could still be with him, but you had help there for those moments that you needed, that we needed help, and that just isn't available. They don't have the space to do it or the personnel.” [1923-06] | 5 |
| *COVID (limited access during pandemic) | “I'm very upset with the facility because during COVID outbreak, I wasn't able to see him for about 10 days. And when I did see him after those 10 days, he had lost like, I don't know, 40 pounds it seemed like, so thin. And I was just frustrated because they were continuing to try to feed him solid foods, but he was barely eating anything. They should have made the decision, I felt like, to switch him to the Ensures, the Boost, or whatever the protein drinks.” [1927-02]  “because of the pandemic, I didn't really want to put him someplace because I was afraid he'd for sure catch it if he was in the home. But I wanted options, I wanted to know that I didn't have to do it all myself because I was seriously sleep deprived.” [1927-44]  Uh, I'm confident that she did, she was in a facility, she was in memory care. Uh, one of the things that makes me confident that she did, I mean the last time that she smiled, she had been, uh, she had been complaining of abdominal pain, uh, severe, this was on a Thursday. Uh, over my objections, the facility had her sent to the ER, uh, the doctor, the attending physician at the ER was the one that recommended hospice. But, uh, I met her at the ER and transported her back to the facility and, uh, when we got to the facility, I let them know we were there so they could send some people out help her out of the, out of my car and onto her own chair and into the facility, they were under COVID restructure at the time, so I couldn't accompany her. But when she saw the caregivers coming to get her, uh, and specifically the director of this facility, uh, she broke up in, into a big smile, the smile that I used to get when I would come to see her. Uh, so I know she was well cared for and they helped her with when she had her anxiety or whatever. So that that smile, that was last time I saw the smile. [1927-54] | 3 |
