# Supplementary material for: End-of-life experiences in individuals with dementia with Lewy bodies and their caregivers: A mixed-methods analysis
Source: PLoS One. 2024 Aug 29;19(8):e0309530. doi: 10.1371/journal.pone.0309530 (PMC11361593; doi:10.1371/journal.pone.0309530)
Supplement: Attachment — Submitted filename: PACE-DLB EOL Resp to Rev 7.11.24.docx [file pone.0309530.s005.docx]

**Response to Reviewers for PONE-D-24-08238, End-of-Life Experiences in Individuals with Dementia with Lewy Bodies: a Mixed-Methods Analysis**

**Reviewer 1**

Thank you for submitting an interesting manuscript that explores caregiver-reported end-of-life experiences in DLB. Please consider the following suggestions for improvement by section.

Title:
- Although the title references “End-of-Life Experiences in Individuals with Dementia with Lewy Bodies,” the majority of data are caregiver-reported and may not accurately reflect the patient experience. To avoid confusion, consider incorporating caregivers into the title (e.g., Caregiver-Reported End-of-Life Experiences in Individuals with DLB, End-of-Life Experiences among DLB Patients and Caregivers, Patient and Caregiver Experiences at End-of-Life in DLB, etc.)

**Author Response:** We have changed the title to “End-of-Life Experiences in Individuals with Dementia with Lewy Bodies and their Caregivers: a Mixed-Methods Analysis.”

Abstract:
- In the methods section, please clarify what data was collected from patients, if any

**Author Response:** We have revised the methods section in the abstract to read, “Dyads of individuals with moderate-advanced DLB and their primary informal caregivers were recruited from specialty clinics, advocacy organizations, and research registries and followed prospectively every 6 months. The current study examines results of caregiver study visits 3 months after the death of the person with DLB. These visits included the Last Month of Life survey, study-specific questions, and a semi-structured interview querying end-of-life experiences.” (We also made edits to comply with the 300 word count limit.) Data was from the post-death visit completed by the caregiver 3 months after the death of the person with DLB. We have also added this clarification to the last paragraph of the introduction (page 5): “The current mixed-methods analysis investigates end-of-life experiences for the first 50 study participants who died during the study *as reported by caregivers at the 3-month post-death visit*.”

Introduction:
- You state: “Reported survival is longer in pathologic cohorts.” Please define the term “pathologic cohorts” for those that are unfamiliar. I assume these were patients who underwent post-mortem examination, but this was not immediately clear.
**Author Response:** We have changed the wording to, “Reported survival is longer in pathologic cohorts (i.e., with post-mortem examination) [3]…” (page 4)

- You state, “Allowing for multiple categories, the most common cause of death in DLB is failure to thrive (65%), followed by pneumonia/swallowing difficulties (23%), medical conditions other than pneumonia (19%), and falls or complications from a fall (10%). How big was this cohort? The denominator will help put these figures into context, as death certificates are often unavailable when using national data.

**Author Response:** We have revised this sentence to read, “Based on a prior survey (n=658), the most common cause of death in DLB is failure to thrive (65%), followed by pneumonia/swallowing difficulties (23%), medical conditions other than pneumonia (19%), and falls or complications from a fall (10%) (multiple categories allowed) [4].” (page 4)

- Similarly, when you state “In a 2017 survey conducted through the Lewy Body Dementia Association, only 40% of family members of individuals with DLB said their physician discussed what to expect at the end of life in DLB,” what was the sample size?

**Author Response:** We have added the “n” from the prior study so that the sentence now reads: “In a 2017 survey conducted through the Lewy Body Dementia Association (n=658), only 40% of family members of individuals with DLB said their physician discussed what to expect at the end of life in DLB. Only 22% reported this was discussed to a helpful degree [4].” (page 4)

- You report that “The current mixed-methods analysis investigates end-of-life experiences for the first 50 study participants who died during the study.” Why are you reporting these data separately from the parent study?

**Author Response:** The parent longitudinal study is looking at patient and caregiver experiences in moderate-advanced DLB, which involves a broad assessment every 6 months. The current analysis focuses only on results of the 3-month post-death caregiver experience, which is its own study aim. Additionally, the current mixed-methods analysis reports specifically on caregivers who completed the original semi-structured interview, which subsequently changed since saturation of themes was reached. We have revised the study participant section in the methods to clarify this: “The current analysis includes the first 50 dyads with a post-death study visit. Because saturation of themes was reached with this cohort, the interview questions subsequently changed. Thus, the current analysis includes visits relating to the original semi-structured interview. Included post-death visits reflect participants who died after the baseline visit and before the 6-month follow up and participants who had multiple study visits prior to death.” (page 6) We also added this information: “The semi-structured interview changed after this time due to saturation of themes for all interview questions.” (page 7) To the study visits and measures section, we added the clarification “Visits occurred every 6 months until the death of the person with DLB. A post-death visit was completed with the caregiver approximately 3 months after the death of the person with DLB *and this visit formed the basis for the current analysis*” (page 7, italics = new).

Methods:
- The inclusion criteria includes persons with DLB expected to live at least 6 months. How was this determined?

**Author Response:** We have revised this to clarify: “…5) person with DLB expected to live at least 6 months (by clinician or participant estimation)…” (page 6)

Results:
- You present mean years between diagnosis and death. It would be helpful to present data on disease duration (symptom onset to death) if available.

**Author Response:** We only collected year of diagnosis, not year of symptom onset.

- There were 37 male patients and 36 patients who identified as male. Was one patient transgender? Consider changing sex and gender to “biological sex” and “gender identity” to avoid confusion.

**Author Response:** We asked sex and gender separately, but did not ask participants on additional details how they view their identity. It is correct that one person identified a different gender than sex. We have changed the terms to “Biologic Sex” and “Gender Identity” as requested (Table 1).

- Caregivers most commonly reported that individuals with DLB died from the DLB itself. How? Please elaborate.

**Author Response:** This was caregiver-reported and details were not provided. We edited the sentence to read, “Caregivers most commonly reported that individuals with DLB died from the DLB itself (21, 42%; exact details unknown) or failure to thrive (9, 18%).” (page 12)

- In the section “Healthcare factors associated with Positive or Negative Experiences,” please describe the negative factors in more detail. For example, what were the negative experiences surrounding hospice care?

**Author Response:** We have made revisions throughout this section (page 25-26) to specifically call out the negative experiences. Examples of calling out the negative experiences, “Lacking communication was a negative experience that made it harder for caregivers to coordinate care.” (page 25). “The degree of help coordinating care at the end of life was a driver of the end-of-life experience, both positively and negatively, with lacking coordination increasing caregiver burden: “the nurses in a way were more of a nuisance because of the constant changing times and days” (1927-59, wife).” (page 25) “Staff turnover and insufficient staffing also negatively affected end-of-life experiences for individuals with DLB living in facilities and their families. Caregivers described negative experiences with both under- and over-medication of the individual with DLB at the end of life.” (page 26)

Discussion:
- A 90% hospice utilization rate is quite high, even if initiated later than appropriate. Could the high utilization rate be based on referral patterns from outpatient doctors (more than 50% saw a DLB specialist) or due to participant bias, given that data were drawn from patients who were willing to participate in end-of-life care research? I would add this to study limitations since this affects generalizability.

**Author Response:** We have added this to the limitation section (page 30-31): “While hospice use was sometimes brief, overall hospice utilization was high (90%). This may reflect the fact that 50% of participants reported DLB, dementia, or movement disorders specialty care and/or that caregivers enrolled in the study were already highly engaged in their loved one’s care and/or advocacy organizations. This also has implications for generalizability.”

General:
- Writing could be more concise, particularly in the introduction

**Author Response:** We have edited the introduction to take out some sentences that were less important (e.g., we removed “DLB is one of two dementias under the Lewy body dementia umbrella”) and to make other sentences less wordy.

**Reviewer 2**

The paper is good and interesting but needs minor revision. See my comments below

Abstract: It does not have conclusion which should be strictly based on the results. Discussion should be deleted from the abstract.

**Author Response:** We have changed the header of the final section from discussion to conclusions. We have also edited the wording to show the conclusions tie to the results. At the same time we have edited other parts of the abstract to make sure it is not over the 300 word limit.

Introduction: DLB expand to full at the beginning of the statement
**Author Response:** Dementia with Lewy bodies is written out in full in the first sentence of the introduction.

Study visits and measures: Correct priori. Please be specific of what you did in the research-visits were completed over multiple. days if needed.

**Author Response:** We are uncertain what “priori” needs to be corrected. We reviewed all occurrences of “priori” and “prior” in the manuscript. The sentence “Study visits were designed for virtual and in-person completion given a priori plans for inclusion of a virtual cohort” (page 7) appears correct and this is the only occurrence of “priori.” Where “prior” is used this is what is intended (i.e., we did not use “prior” when intending “priori”). We have removed the sentence about completing research visits over multiple days because this was not relevant for the post-death caregiver study visits. All post-death caregiver visits except one were completed in one sitting.

Data Availability: Indicate the author to contact for data availability

**Author Response:** We have added “by the corresponding author” (page 9).

Sample size of 50. It is small, you would have followed more subjects.

**Author Response:** The parent PACE-DLB is studying more participants. The current analysis looks at the first 50 participants who died because saturation of themes for the interview questions was achieved with this sample size (which is actually larger than most qualitative studies). We have now clarified this in a couple places: “The current analysis includes the first 50 dyads with a post-death study visit. Because saturation of themes was reached with this cohort, the interview questions subsequently changed. Thus, the current analysis includes visits relating to the original semi-structured interview. Included post-death visits reflect participants who died after the baseline visit and before the 6-month follow up and participants who had multiple study visits prior to death.” (page 6) We also added this information to the methods: “The semi-structured interview changed after this time due to saturation of themes for all interview questions.” (page 7)

Conclusion: Remove the table you mentioned on the conclusion. The conclusion is too long. Kindly summarise the key findings and implications.

**Author Response:** We have deleted Table 4 as requested. We have also edited the paragraph to make it more concise.
